# The Saudi Initiative for Asthma

**DOI:** 10.4103/1817-1737.56001

**Published:** 2009

**Authors:** Mohamed S. Al-Moamary, Mohamed S. Al-Hajjaj, Majdy M. Idrees, Mohamed O. Zeitouni, Mohammed O. Alanezi, Hamdan H. Al-Jahdal, Maha Al Dabbagh

**Affiliations:** *Department of Medicine, College of Medicine, King Saud bin Abdulaziz University for Health Sciences, Riyadh, Saudi Arabia*; 1 *Department of Respiratory Division, Department of Medicine, Medical College, King Saud University, Riyadh, Saudi Arabia*; 2 *Department of Pulmonary Division, Department of Medicine, Military Hospital, Riyadh, Saudi Arabia*; 3 *Department of Medicine, King Faisal Specialist Hospital and Research Center, Riyadh, Saudi Arabia*; 4 *Department of Pulmonary Section, Department of Pediatrics, King Fahd Armed Forces Hospital, Jeddah, Saudi Arabia*

**Keywords:** Asthma, guidelines, Saudi Arabia

## Abstract

The Saudi Initiative for Asthma (SINA) provides up-to-date guidelines for healthcare workers managing patients with asthma. SINA was developed by a panel of Saudi experts with respectable academic backgrounds and long-standing experience in the field. SINA is founded on the latest available evidence, local literature, and knowledge of the current setting in Saudi Arabia. Emphasis is placed on understanding the epidemiology, pathophysiology, medications, and clinical presentation. SINA elaborates on the development of patient-doctor partnership, self-management, and control of precipitating factors. Approaches to asthma treatment in SINA are based on disease control by the utilization of Asthma Control Test for the initiation and adjustment of asthma treatment. This guideline is established for the treatment of asthma in both children and adults, with special attention to children 5 years and younger. It is expected that the implementation of these guidelines for treating asthma will lead to better asthma control and decrease patient utilization of the health care system.

Asthma is a common chronic disorder of the airways, characterized by variable reversible and recurring symptoms related to airflow obstruction, bronchial hyperresponsiveness, and an underlying inflammation. It is one of the most common chronic diseases in Saudi Arabia, affecting more than 2 million Saudis.[1] Its impact is manifested in patients, their families, and the community as a whole in terms of lost work and school days, poor quality of life, frequent emergency department visits, hospitalizations, and death cases.[2] Therefore, international guidelines have been developed to help the physicians to manage asthma in a better way and deal with different presentations and situations using the best available evidence.[3]

The Saudi Thoracic Society (STS) is committed to a long-term enhancement plan for the best practice in the asthma field by creating asthma guidelines, periodic scientific meetings, frequent asthma courses, and educational brochures. With massive influx of knowledge in the literature, the STS has taken the lead to create the Saudi Initiative for Asthma (SINA) group with the objective to have easy guidelines to follow, yet simple to understand updated and carefully prepared for use by the nonasthma specialists including primary care and general practice physicians.[4] The SINA panel is a group of local experts with respectable academic backgrounds and long-standing experience in the field.

## Methods

The initial draft was based on two existing international guidelines developed by the Global Initiative for Asthma (GINA) and the National Asthma Education and Prevention Program (NAEPP).[5,6,7,8] Customization was based on reviewing the available local literature and the current setting in Saudi Arabia. Consensus among the SINA panel was followed whenever there was a lack of evidence in the form of randomized controlled trials or nonrandomized studies.[9] The system used the following to describe the level of evidence:

- Evidence Category A: Randomized controlled trials with rich body of data.
- Evidence Category B: Randomized controlled trials with limited body of data.
- Evidence Category C: Nonrandomized trials and observational studies.
- Evidence Category D: SINA Panel consensus judgment. This category is used only in cases where the provision of some guidance was deemed valuable, but the clinical literature addressing the subject was insufficient to justify placement in one of the other categories.

Each section was written by one of the authors and internally reviewed by at least two other members. The SINA panel conducted round-table discussions on frequent occasions and jointly reviewed it. A panel of international experts reviewed the guidelines and their recommendations were thoughtfully considered (refer to acknowledgment section). The expected outcome will lead to a safe high-quality patient care.

## Epidemiology of Asthma

Asthma is one of the most common chronic illnesses in Saudi Arabia and local reports suggest that the prevalence of asthma is increasing.[10] Despite the abundance of high-caliber medical services and the availability of international guidelines, recent studies have shown that the burden of asthma might be significantly higher than previously estimated.[11,12] Poor knowledge, fear of use of new drugs, and lack of awareness of the importance of control of the disease are common among primary care physicians caring for asthma patients in the Kingdom of Saudi Arabia.[13] These are important factors that likely contribute to the magnitude of this burden.[14] Consequently, many asthma patients continue to be under-diagnosed, under-treated, and at a risk of acute exacerbations resulting in missing work or school, increased use of expensive acute healthcare services, and reduced quality of life.[15] A recent asthma control survey showed that only 5% were controlled, 31% were partially controlled, and 64% were uncontrolled.[16]

Al-Fryh and colleagues investigated the changing prevalence of asthma in the KSA.[17] Two population segments of school children between the age groups of 8-16 years were studied using an internationally designed protocol in 1986 and 1995. Comparison of the data of Riyadh versus Hail (an inland desert dry environment) and Jeddah versus Gizan (a coastal humid environment) revealed that the prevalence of asthma in similar populations increased significantly from 8% in 1986 to 23% in 1995.[18] The study also revealed increased exposure to environmental factors such as tobacco smoke and indoor animals in Saudi houses.

Other studies in the KSA compared the prevalence of physician-diagnosed asthma among Saudi school boys in Yanbu, an industrial city, to two nonindustrial villages in a cross-sectional study.[19] The prevalence in Yanbu and in the villages of Al-Furash and Al-Gafure was 13.9% and 8%, respectively. Hijazi and colleagues conducted a study on 1,020 urban and 424 rural children of 12-year age group to compare the prevalence of allergic symptoms among those living in urban and rural areas of the KSA and to investigate factors associated with any differences found.[20] The prevalence of allergic symptoms was found to be significantly greater among urban children than the rural ones and more among Saudi children than non-Saudi children.[21] Males were more susceptible to have some respiratory symptoms and females had more eye and skin symptoms. The educational level and occupation of the father did not influence the likelihood of having the symptoms. A study by Al-Kabbaa and colleagues found that 39% of primary care physicians meet the standards of the national guidelines in management of asthma.[22] Furthermore, the overall level of awareness among physicians was low (52%). Their proficiency in general knowledge, diagnosis, classification of severity and management was also low.

## Pathophysiology of Asthma

### Airways inflammation

**Figure 1:**
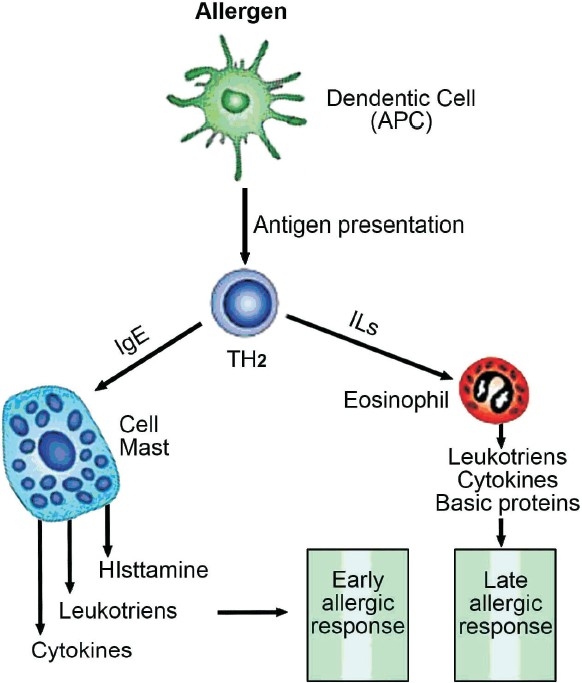
Pathophysiology of asthma APC= Antigen presenting cell, ILs= Interleukins, TH_2_= T-lymphocyte Helper cell 2

Asthma is a complex syndrome characterized by a state of airways hyperresponsiveness (AH) and caused by a multi-cellular inflammatory reaction that leads to airway obstruction [Figure 1].[23] Recruitment and activation of mast cells, macrophages, antigen-presenting dendritic cells, neutrophils, eosinophils, and T lymphocytes result in an inflammatory and cellular infiltration of the airways.[24] Type 2 T-helper cells (Th2) have a major role in the activation of the immune cascade that leads to the release of many mediators such as interleukins (IL)-3, IL-4, IL-5, IL-13, and granulocyte macrophage colony stimulating factor (GM-CSF).[25,26] Some mediators such as IL-4 activate B lymphocytes to produce immunoglobulin E (IgE), and others (for example, IL-3, IL-5, and GM-CSF) are related to the eosinophilic airway inflammation. Severe asthma may present various inflammatory phenotypes, such as persistent eosinophilic bronchitis, neutrophilic infiltration of the airway, and a pauci-granulocytopenic type of inflammation.[27] Such persistent inflammatory process results in airway remodeling with deposition of extracellular proteins, smooth muscle hypertrophy, and increased goblet cell production.[28] Airway epithelium becomes fragile and thin, and the epithelial basement membranes thicken with increased mucus production and endothelial leakage leading to mucosal edema. Mediator-induced abnormalities in the parasympathetic and nonadrenergic noncholinergic nervous systems may also lead to increased bronchial hyperresponsiveness. Recent data has shown that involvement of the upper airways by similar inflammatory reaction is present in almost all asthmatic patients, irrespective of the presence of symptoms of rhinosinusitis. Studies have shown that stimulation of the nose by an irritant instilled in the nose leads to eosinophilic infiltration in the lungs a few hours later. Such coexistence of inflammation in both upper and lower airways led to the suggestion of new terminology called united airway disease. In clinical practice, failure of recognition and treatment of concomitant rhinosinusitis may affect asthma control.[29]

### Airways hyperresponsiveness

Airways hyperresponsiveness (AH) to direct (for example, histamine or methacholine) and indirect (for example, exercise, cold air, mannitol, adenosine monophosphate or isocapnic hyperventilation) challenges is a characteristic of asthma.[30] When asthma symptoms are present, there is a relatively good correlation between the severity of disease and the degree of AH.[31] AH is not a static feature of asthma: it may increase after sensitizing exposures and may decrease after antiinflammatory treatments or a reduction in relevant environmental exposures. Asthma has a variable component related to airway inflammation, and a more refractory component that is largely attributed to underlying airway structural changes that are also known as remodeling.[32]

### Early and late responses

Following presentation of the antigen by dendritic cells in a sensitized patient, certain inflammatory cascades get activated leading to attachment of Immunoglobulin E (IgE) antibodies to inflammatory cells such as mast cells.[33] This activates the inflammatory cells, which respond by degranulating, and hence, liberating various inflammatory mediators responsible for the allergic response. The allergen-induced airway response may be immediate (the early response)—with a fall in expiratory flows within an hour of exposure, or may be delayed (the late response) with the fall in expiratory flows being observed within two and eight hours later. An increase in AH and in the variability of airway obstruction may then occur in the following days, depending on the intensity of the response.[34]

### Airway remodeling

Structural airway changes, may develop even before the disease becomes symptomatic, or in conditions such as allergic rhinitis that are associated with an increased risk of developing asthma. The most prominent of these changes are epithelial damage, subepithelial fibrosis, increased airway vasculature, increases in proteoglycans, and increased smooth-muscle mass. The mucus hypersecretion observed in asthma is related to an increase in the number of secretory glands and cells such as goblet cells. These changes are generally attributed to the underlying inflammatory process, although other mechanisms may play a role.[35] It has been proposed that remodeling may be involved in the development and persistence of asthma, in the accelerated decline in pulmonary function observed in asthma, and in the development of a more ‘fixed’ component of airway obstruction in some asthmatic patients. Although a relationship has been found between the severity of asthma and some of the components of airway remodeling, researchers have not yet been able to adequately distinguish severe asthma from milder forms on the basis of histological features alone.[36] Prevention of airway remodeling has not been well studied, but it is possible that prompts and sustained treatment of asthma with antiinflammatory controller medications and prevention of exacerbations may have a role in prevention or delaying airway remodeling.

## Diagnosis of Asthma

The clinical assessment of asthma includes a detailed history and physical examination supplemented by spirometry, with reversibility testing, to support the diagnosis. To date, there is no single diagnostic test to confirm asthma, although the assessment of AH is helpful in this regard.[37,38] The symptoms of wheezing, cough, shortness of breath, and chest tightness are not specific for bronchial asthma and can be seen with other pulmonary diseases. These symptoms are variable over time and the patient may be entirely asymptomatic between attacks.[39] Symptoms are usually worse at night, particularly in children, and could be provoked by exercise or other triggering factors. Table 1 lists the relevant questions that are commonly considered in history taking.

**Table 1:** Relevant questions in the diagnosis of asthma

Does the patient or his/her family have a history of asthma or other atopic conditions, such as eczema or allergic rhinitis? Does the patient have recurrent attacks of wheezing? Does the patient have a troublesome cough at night? Does the patient wheeze or cough after exercise? Does the patient experience wheezing, chest tightness, or cough after exposure to pollens, dust, feathered or furry animals, exercise, viral infection, or environmental smoke (cigarettes, burning incense “Bukhoor”, or wood? Does the patient experience worsening of symptoms after taking aspirin/nonsteroidal inflammatory medications or use of B-blockers? Does the patient's cold “go to the chest” or take more than 10 days to clear up? Are symptoms improved by appropriate asthma treatment?

Some patients, particularly children, have a cough as the main or the only symptom without wheezing or shortness of breath, which is called cough variant asthma. In this situation, the diagnosis may be confirmed by a positive response to asthma medication. Others may have their asthma induced by exercise only, a condition called exercise-induced asthma (EIA). Symptoms of asthma could be worsened by coexistent gastro-esophageal reflux disease (GERD), rhinosinusitis, or the use of some medications such as beta blockers and nonsteroidal antiinflammatory agents (NSAID), and Aspirin (ASA). Asthma and rhino-sinusitis commonly occurs concomitantly.[40]

Physical examination usually reveals bilateral expiratory wheezing, which may be absent between attacks. Examination of the upper airways is important to look for evidence of allergic rhinitis, such as mucosal swelling, nasal polyps, and postnasal dripping. Other allergic manifestations, such as atopic dermatitis/eczema may also support the diagnosis of allergic asthma.[41] The presence of localized wheeze, crackles, stridor, clubbing, or heart murmurs should suggest alternative diagnoses.[42,43]

Spirometry is necessary to confirm airflow obstruction, assess its severity and to demonstrate significant reversibility [Table 2]. Normal spirometry, including a failure to show reversibility, does not rule out the diagnosis of asthma, as it can be normal with the patient still being symptomatic.[44] Serial peak expiratory flow (PEF) measurement may be helpful in asthma diagnosis and follow-up. Bronchoprovocation testing is another tool used by specialist[9] to role out asthma; however, a trial with inhaled steroids and bronchodilator may be useful in diagnosis.[45]

**Table 2:** Acceptable spirometry and significant bronchodilator response

Proper instructions on how to perform the forced expiratory maneuver must be given to patients, and the highest value of three reading taken. The degree of significant reversibility is defined as FEV1 ≥ 12% and ≥200 ml from the prebronchodilator value.

Chest x-ray is not recommended unless the diagnosis is in doubt, symptoms are not typical, or to rule out other diagnoses. Peripheral eosinophilia and elevated IgE level are supportive of the diagnosis, but are not routinely recommended. Skin testing and radioallergosorbent test (RAST) may be helpful in identifying allergens to which the patient has been sensitized and to develop a strategy for avoiding allergen exposure.[46]

### Special consideration for asthma in children younger than five years

The diagnosis of asthma in early childhood is challenging and has to include assessment of symptoms and physical findings and exercising clinical judgment.[47] Since the use of the label “Asthma” for wheezing of children has important clinical consequences, it must be distinguished from other causes of persistent and recurrent wheezing in this age group.[48] The natural history of wheezing is dependent on age at first presentation; the earlier the onset of a wheeze, the better the prognosis.[49] However, the presence of coexistent atopy is considered a risk factor for persistence of wheezing independent of age when first diagnosed. A family history of atopy and asthma and maternal atopy are strongly associated with persistent childhood asthma.[50]

Three categories of wheezing have been described in children of five years and younger [51,52,53]:

- *Transient early wheezing*: It is often outgrown in the first three years, when symptoms begin and end before the age of three. This is often associated with prematurity and parental smoking.
- *Persistent early-onset wheezing:* Children in this category begin to have symptoms before the age of three and continue beyond the age of six. These children typically have recurrent episodes of wheezing associated with acute viral respiratory infections and have no evidence of atopy.
- *Late-onset wheezing/asthma:* Symptoms begin after the age of three and persist throughout childhood and into adult life. Typically, the patient has an atopic background, often with eczema, and his airway pathology shows characteristic features of asthma.

In the children of five years and below, no tests can diagnose asthma with certainty. Lung function testing, and especially the assessment of airway hyperresponsiveness, is not very helpful in diagnosing asthma in this age group.[54] Skin prick testing is less reliable for confirming atopy in infants. However, a chest radiograph may help to exclude structural abnormalities of the airway.[55] A trial of treatment with short-acting bronchodilators and inhaled corticosteroids (ICS) for at least 8 to 12 weeks may provide some guidance as to the presence of asthma.[56]

## Medications Used for the Treatment of Asthma

The objective of asthma treatment is to achieve and maintain control of the disease. Medications used to treat asthma can be classified as controllers or relievers. *Controllers* are medications taken daily on a long-term basis to keep asthma under clinical control mainly through their antiinflammatory effects.[57] *Relievers* are medications used on an “as-needed basis” that act quickly to reverse bronchoconstriction and relieve symptoms.

### Controller medications

*Inhaled cortico steroids:* ICS are currently the most effective antiinflammatory medications for the treatment of asthma (**Evidence A**).[58,59] They reduce symptoms, improve the quality of life, improve lung function, decrease airway hyperresponsiveness, control airway inflammation, reduce frequency and severity of exacerbations, and reduce asthma mortality.[60] When they are discontinued, deterioration of clinical control follows within weeks to months in most patients.[61] ICS differ in their potency and bioavailability.[62] Most of the benefits from ICS are achieved in adults at relatively low doses [Tables 3 and 4].[63] Increasing to higher doses may provide further benefits in terms of asthma control but increases the risk of side effects.[64] As tobacco smoking reduces the responsiveness to ICS, higher doses may be required in patients who smoke.[65] To reach control, add-on therapy with another class of controller is preferred to increasing the dose of ICS (**Evidence A**);[66,67] however, some patients with severe asthma may benefit from long-term treatments with high doses of ICS. The clinical benefits of intermittent systemic or ICS for children with infrequent viral induced wheezes remain controversial.[68] While some studies in older children have found small benefits, a study in young children found no effects on wheezing symptoms. There is no evidence to support the use of low-dose maintenance inhaled ICS for preventing transient wheezing in childhood.[69] Though low-medium dose ICS may affect growth velocity, this effect is clinically insignificant and may be reversible.

**Table 3:** List of equipotent daily doses in micrograms of the ICS available in Saudi market for adults

Drug	Low dose	Medium dose	High dose
Beclomethasone	200–500	>500–1000	>1000–2000
Budesonide	200–400	>400–800	>800–1600
Fluticasone propionate	100–250	>250–500	>500–1000
Ciclesonide	80–160	>160–320	>320–1280

**Table 4:** List of equipotent daily doses in micrograms of the ICS available in the Saudi market for children

Drug	Low dose	Medium dose	High dose
Beclomethasone	100–200	>200–400	>400
Budesonide	100–200	>200–400	>400
Fluticasone	50–100	>100–200	>200

Inhaled Cortico Steroids are generally safe and well-tolerated. Local adverse effects can occur and include: oropharyngeal candidiasis, and dysphonia. With metered dose inhalers (MDI), these effects may be reduced by using spacer devices. Mouth washing after inhalation may reduce oral candidiasis.[70] Systemic side effects are occasionally reported with high doses and long-term treatment. The small risk of adverse events from the use of ICS is well balanced by their efficacy.[71] Some studies have shown that ciclesonide has relatively lower local and systemic side effects.[72,73] To date, ciclesonide has not been approved for children by the FDA; its safety and effectiveness has not been established in children below 12 years.

*Long-acting inhaled B2-agonists:* Long-acting inhaled B2-agonists (LABAs), including formoterol and salmeterol, should not be used as mono-therapy in asthma (**Evidence A**).[74] In fact, studies show that it is harmful to use them alone to control asthma. Combination with ICS improves symptoms, decreases nocturnal asthma, improves lung function, decreases the use of rescue rapid-onset inhaled B2-agonists, reduces the number of exacerbations, and achieves clinical control of asthma in most patients, more rapidly, at a lower dose of ICS (**Evidence A**).[75,76] Fixed combination inhalers are available in the form of fluticasone and salmeterol (Seretide) or budesonide and formoterol (Symbicort). They are considered more convenient for patients. They increase compliance, and ensure that LABA is always accompanied by ICS. Although salmeterol and formoterol provide a similar duration of bronchodilation and protection against bronchoconstriction, formoterol has a more rapid onset of action than salmeterol.[77] Therefore, combination inhalers containing formoterol and budesonide may be used for both rescue and maintenance of control.[78] LABA provides longer protection to prevent exercise-induced bronchospasm than short-acting inhaled B2-agonists (SABA).[79] In children of five years and above, formoterol combined with budesonide, are used as controller and rescue medications.[80] Their side effects are limited to tachycardia, tremor, headaches, muscle cramps, and rarely hypokalemia. Regular use of LABA may lead to a reduction in these side effects.[81] Rarely patients develop a tolerance to LABAs. The effect of LABA products has not been adequately studied in children of five years and below.

*Leukotriene modifiers:* Leukotriene modifiers (LTRA) reduce airway inflammation and improve asthma symptoms and lung function but with a less consistent effect on exacerbations, especially when compared to ICS.[82] They may be used as an alternative treatment to ICS for patients with mild asthma, especially in those who have clinical rhinitis.[83] Some patients with aspirin-sensitive asthma respond well to the LTRA. However, when used alone as a controller, their effects are generally less than that of low dose ICS. When added to ICS, LTRA may reduce the dose of ICS required by patients with uncontrolled asthma, and may improve asthma control.[84,85] LTRA are generally well-tolerated. There is no clinical data to support their use under the age of six months.

*Theophylline:* Theophylline is a weak bronchodilator with modest antiinflammatory properties.[86] It may provide benefits as an add-on therapy in patients who do not achieve control with ICS alone, but is less effective than LABA and LTRA. Theophylline is not recommended for use as monotherapy in asthma treatment.[87] Recent data has shown that low-dose theophylline may have an important role in improving steroid resistance in patients with severe asthma requiring high dose ICS through activation of certain down-regulated pathways, such as histone deactylases.[88] Their side effects include gastrointestinal symptoms, cardiac arrhythmias, seizures, and even death. Nausea and vomiting are the early symptoms of toxicity. Liver disease, congestive heart failure, some quinolones, and some macrolides increase the risk of toxicity. Use of lower doses may decrease side effects.[87]

*Anti-IgE:* Anti-IgE (Omalizumab) use is indicated for patients of 12 years and above with severe allergic asthma uncontrolled on high dose ICS and other controllers (**Evidence A**).[89] As this drug is expensive and requires careful monitoring, it should only be used by a specialist.[90] The side effects include pain and bruising at injection site and very rarely anaphylaxis (0.1%).[90]

*Oral B2-agonists:* The side effect profile is much higher than that of inhaled B2-agonists. Therefore, their use is highly discouraged.[87]

*Systemic corticosteroids:* Long-term oral glucocorticosteroid therapy (excluding short courses for acute attacks of asthma for a period of 1-2 weeks) may be required to control the difficult asthma despite maximum standard therapy. The dose should be reduced to the lowest and other controllers should be maximized to minimize the side effects. Its use is limited by the risk of significant adverse effects.[91] Use of intramuscular long-acting steroids is highly discouraged because of the increased risk of side effects. The side effects include: osteoporosis, hypertension, diabetes, adrenal insufficiency, obesity, cataracts, glaucoma, skin thinning, and muscle weakness. Withdrawal can elicit adrenal failure. In patients prescribed long-term systemic corticosteroids, prophylactic treatment for osteoporosis should be considered.[92]

### Reliever medications

Relievers are medications used on as-needed basis that act quickly to reverse bronchoconstriction and relieve symptoms.

*Rapid onset inhaled B2-agonists:* SABA such as salbutamol is the medication of choice for relief of symptoms of acute exacerbations of asthma and for the pretreatment of exercise-induced bronchoconstriction.[93,94] Formoterol is used for symptom relief because of its rapid onset of action.[95] However, when it is used for maintenance therapy, it should always be given with ICS. Increased use, especially daily use, is a warning of the deterioration of asthma control and indicates the need to reassess treatment. Their side effects are systemic such as tremor and tachycardia. Regular long-term use of SABA is not recommended.[96]

*Anticholinergics:* Anticholinergics, in asthma, are less effective than SABA. However, when used in combination with SABA in acute asthma, they have an additional effect. Benefits in long-term management of asthma have not been established.[97] It can also be an alternative bronchodilator for patients who experience such adverse effects as tachycardia, arrhythmia, and tremor from rapid acting B2-agonists. Their side effects include dryness of the mouth and a bitter taste.

*Theophylline:* There is no evidence supporting the routine use of theophylline in treating acute asthma and its use is discouraged.

## Approach to Asthma Management

The long-term goal of asthma therapy is to achieve and maintain asthma control by utilizing pharmacological and non-pharmacological measures [Table 5]. This should lead to utilization of the least possible dose of medications in order to minimize the risk of side effects.

**Table 5:** The long-term goals of asthma management

Control asthma symptoms (cough, wheezing, or shortness of breath) Infrequent and minimal use (≤2 days a week) of reliever therapy Maintain (near) normal pulmonary function Maintain normal exercise and physical activity levels Prevent recurrent exacerbations of asthma, and minimize the need for emergency room visits or hospitalizations Optimize asthma control with the minimal dose of medications Reduce mortality Optimize quality of life

### Nonpharmacological measures

#### Developing partnership with the patient

The development of partnership between the patient and healthcare professionals leads to enhancement of knowledge, skills, and attitude toward understanding asthma and its management (**Evidence A**).[95] Based on agreed goals of management, guided-written self-management plan is offered to the patient. A wide variety of plans are available which vary from patient-based to physician-based plans. This should be reflected positively on patient adherence, which is a major issue in the management. Factors leading to nonadherence may be related to poor inhaler technique, a regimen with multiple drugs, concern regarding side effects from the drugs, or the cost of medications.[98,99,100] Other factors include: lack of knowledge about asthma, lack of partnership in the management, inappropriate expectations, underestimation of asthma control, use of unconventional therapy and cultural issues.[101,102]

#### Asthma education

The goal of asthma education is to provide the person with asthma (or parents of a child) adequate training to enhance their knowledge and skills to be able to adjust treatment according to guided self-management (**Evidence A**).[103,104] Education should include knowledge about asthma and skills related to the devices. Asthma education should be conducted by a well-trained healthcare worker with good communication skills, who is able to create an interactive dialogue in a friendly environment. With the availability of appropriate information, patients will be encouraged to continue on the management plan and reassured about the control of the disease.[105] It is essential to get the feedback of the patient in order to maintain a bidirectional rapport and optimum environment. It has been documented that a well-structured asthma education program will improve quality of life, reduce cost and utilize of health care resources.[106,107] Asthma education should be tailored based on the available setting [Table 6].

**Table 6:** Outcomes of asthma education program

Creation of partnership between the patient and the healthcare worker Understanding clinical presentation of asthma and methods of diagnosis Ability to differentiate between “relievers” and “controllers” medications and their appropriate indications Recognition of potential side effects of medications and the appropriate action to minimize them Performance of the proper technique of different inhaler devices Identification of symptoms and signs that suggest worsening of asthma and the appropriate action to be taken Understanding the approach for monitoring asthma control Recognition of the situations that need urgent medical attention Ability to use a written self-management plan

#### Written action plan for asthma

It is considered an integral part of management for patients with asthma and his/her doctor. It helps to recognize loss of control of asthma and gives clear instructions for early intervention to prevent asthma attacks. Use of an asthma action plan leads to better control in both children and adults. The asthma action plan may be based on symptoms or PEF measurements.[108] Regular review of the asthma action plan is important as a person's level of asthma control may change over time. If a patient has an exacerbation, he/she should be assessed for the has effective use of their action plan.

#### Identify and reduce exposure to risk factors

Measures to prevent or reduce exposure to risk factors should be implemented wherever possible. There are different triggers leading to asthma exacerbations, which may include: allergens, viral infections, pollutants, drugs, and occupational agents. These factors can be classified as indoor or outdoor allergens and occupational sensitizers.

*Indoor allergens and air pollutants:* There is a wide spectrum of indoor allergens that includes domestic mites, furred animals, cockroaches, and fungi.[109,110] Most of the interventions to reduce exposure to these triggers, are helpful, but likely alone, will not help to achieve asthma control. The most important indoor air pollutant is related to tobacco exposure.[111] Measures to avoid tobacco exposure will lead to better asthma control and avoidance of long-term lung function impairment.

*Outdoor allergens:* Outdoor allergens such as pollens and molds are impossible to avoid completely. Exposure may be reduced by closing windows and doors, remaining indoors during dust storms and initial raining seasons and using air conditioning if possible. It is recommended to avoid outdoor strenuous physical activities in cold weather, low humidity, or high air pollution.[112]

*Occupational exposures:* Whenever an occupational sensitizer is identified, it is advisable to keep the affected person away from that environment. The earlier the removal of this sensitizer takes place, the higher the chance of complete recovery from occupational asthma.

*Food and drugs:* Food and food additives are uncommon triggers of asthma. Avoidance cannot be recommended until it is documented by a specialist. However, certain drugs whenever identified should be avoided e.g., beta blockers.

*Influenza vaccination:* Annual influenza vaccination is advised for individuals with severe asthma. Other asthmatics may benefit from vaccination, although it does not appear to protect from asthma exacerbations or improve asthma control (**Evidence B**).[113]

### Pharmacological measures

#### Principles for optimal asthma management

The principles of optimal asthma management should follow assessment of asthma control. The severity index is not recommended to classify asthma as it is not practical due to the change of severity over time (**Evidence D**).[114]

#### Initiation of treatment

**Figure 2:**
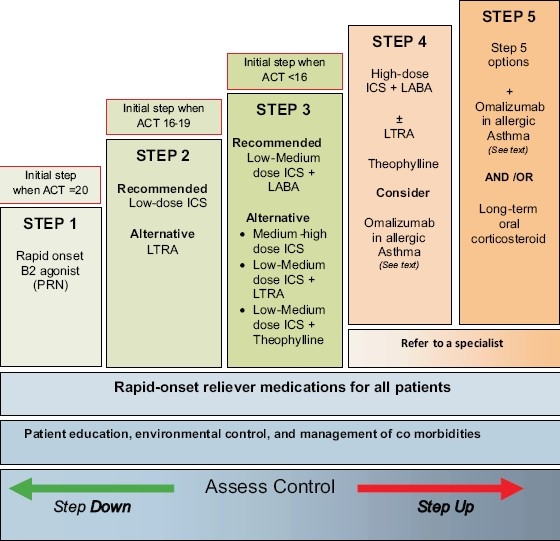
Stepwise approach for managing asthma in adults

**Figure 3:**
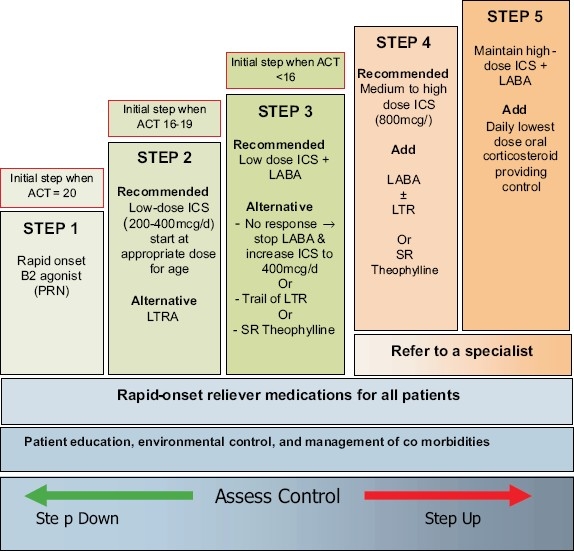
Stepwise approach for managing asthma in children between the age group of 5-12 years

**Figure 4:**
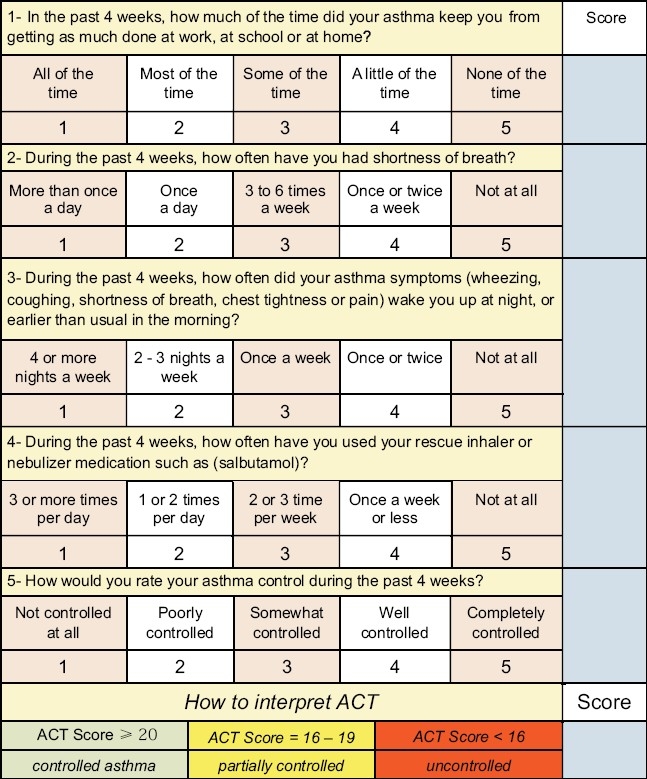
Asthma control test

For patients who are *not* currently taking long-term controller medications, step 2 is recommended as the initial step [Figures 2 and 3]. If the initial symptoms are more frequent, treatment is recommended at step 3. Never the less, some patients have little symptoms where SABA on as needed bases is enough (**Evidence D**). The consensus among SINA panel is to simplify the approach to initiate asthma therapy by using ACT [Figure 4].[115] A score of ≥20 will lead to step 1, 16-19 to step 2, and less than 16 to step 3 (**Evidence D**).

#### Adjustment of treatment

The level of asthma control is categorized into: controlled, partially controlled, and uncontrolled [Table 7] according to symptoms, relievers use, pulmonary function, exacerbation and a validated asthma questionnaire, such as asthma control test (ACT). The SINA expert panel recommends the utilization of ACT to assess control as it is validated, practical, and measurable (**Evidence D**) [Figure 4].[116,117] ACT is a brief patient-administrated questionnaire that can be measured and followed over time. It is a five-item multidimensional instrument to assess asthma control. A score of ≥20 indicates controlled asthma, 16-19 partially controlled, and less than 16 uncontrolled.

**Table 7:** Assessing asthma control in adults

Component of control	Classification of asthma contro		
	
	Controlled	Partially controlled	Uncontrolled
Symptoms	≤2 days/week	>2 days/week	Throughout the day
Nighttime awakenings	≤2 times/month	1-3 times/week	≥4 times/week
Effect on daily activities	None	Some limitations	Extremely limited
Rapid-onset B2-agonist for symptoms relief (not including preexercise prophylaxis)	≤2 days/week	>2 days/week	Several time/day
FEV1 or peak flow	>80% of predicted/ personal best	60-80% of predicted/ personal best	<60% of predicted/ personal best
Validated questionnaire: ACT score	≥20	16-19	<16
Exacerbation (requiring oral steroids or hospitalization) (needs more discussion)	0	0-2/year	≥2/year

A stepwise approach to the therapy is used to achieve asthma control. The steps (1-5) of care for managing asthma are shown in Figures 2 and 3. Therapy is stepped up to achieve control, and stepped down for patients who have maintained control for a sufficient length of time[118]. It is important to determine the minimal amount of medications required to maintain control and reduce the risk of side effects. As most asthma patients have concomitant rhinosinusitis that affect their control, treatment of this condition will improve asthma control (**Evidence C**). This includes nasal steroids, LTRA, and antihistamines. Coexisting sinusitis should be treated appropriately.

#### Maintaining control of asthma

Regular follow-up by a healthcare worker is essential. Depending on the level of asthma control, it is recommended to have a follow-up interval every one to six months (**Evidence D**).[104] The follow-up should include monitoring and review of the patient's written asthma action plan, medications, patient's behaviors, and the side effects of medications. Once asthma is well-controlled and the control is maintained for at least three months, a reduction in pharmacologic therapy (a step-down) is recommended to reach the minimum therapy level that can maintain a good control of asthma and minimize the side effects (**Evidence D**). The following are recommended:

- Reduction in therapy should be gradual and closely monitored based on clinical judgment of the individual patient's response to therapy (**Evidence D**).
- If the patient is on ICS as monotherapy, the dose of ICS may be reduced by 25-50% every three months to the lowest dose possible that is required to maintain control (**Evidence B**),[119] and then shifted to single daily dose (**Evidence A**).[120] It should be explained to the patient that asthma control may deteriorate if abruptly discontinued.
- If the patient is on a combination of ICS and LABA, LTRA, or other controllers, then tapering ICS to the lowest possible dose (**Evidence B**).[121] If control achieved, LABA or LTRA may be discontinued or combination therapy shifted to once daily (**Evidence D**).[122]
- For significant side effects, consider a change in therapy, reduction in the dose or frequency of ICS (if possible), advice for vigorous mouth wash after inhalation, use of spacer (in concomitant with MDI devices), or use of appropriate local antifungal therapy for severe oral thrush.[123]

If asthma control is not maintained at any step of therapy, the following actions are recommended:

- Patient's adherence and technique in using medications correctly should be assessed as follows:
  - Medications and their doses currently taken.
  - Selection of the appropriate device and appropriate prescription of spacer with MDI devices.
  - Inhaler devises technique.
  - Occasions when medication skipped per week.
  - Problems and difficulties facing the patient after taking the medications e.g., cost, time, lack of perceived need… etc.
  - Patient's concerns about asthma medications.
- Other factors that affect control should be carefully evaluated and ruled out. They include:
  - Unrecognized rhinitis or rhinosinusitis.
  - A coexisting condition (for example, GERD).
  - An environmental or occupational exposure.
  - The administration of new medications (for example, beta blockers or nonsteroidal antiinflammatory agents).
  - Psychosocial problems such as depression.
  - In some cases, alternative diagnoses, such as vocal cord dysfunction (VCD), should be considered.

If the above are ruled out, a step-up in therapy is recommended. However, in severe cases a course of systemic steroid may be indicated to regain asthma control.

Referral to an asthma specialist for consultation or comanagement are recommended in these situations:

- There are difficulties achieving or maintaining control of asthma.
- Immunotherapy or Omalizumab is being considered.
- The patient requires step 4 care or higher; or
- The patient has had an exacerbation requiring a hospitalization.

## Treatment (Pharmacologic) Steps

The recommendations for treatment steps are intended to be general guidelines for assisting, not replacing, clinical decision making.

### Treatment at Step 1

The symptoms are usually mild and infrequent [Figures 2 and 3]. However, some patients may experience sudden, severe, and life-threatening exacerbations. It is essential to treat these exacerbations accordingly. This category should follow step 1 by considering rapid onset B2-agonist (SABA or rapid-onset LABA) to be taken “as needed” to treat symptoms (**Evidence A**).[94,95,96] It is usually sufficient therapy for this level. However, if treatment frequency increases to more than two days a week, then the patient should be treated as partially controlled asthma (see below).

### Partially controlled or uncontrolled asthma (Treatment at Steps 2-5)

For partially controlled asthma or uncontrolled asthma, the following general roles are recommended [Figures 2 and 3]:

- Daily long-term controller medication is needed. ICS are considered as the most effective controller (**Evidence A**).[59,60]
- Relievers or rescue medications must be available to all patients at any step. SABA or rapid onset LABA should be taken as needed to relieve symptoms. Increasing use of reliever treatment is usually an early sign of worsening asthma control (**Evidence A**).[94,95]
- Treat patients who may have seasonal asthma as having uncontrolled asthma during the season at step 1 for the rest of the year (**Evidence D**).
- Patients who had two or more exacerbations requiring oral corticosteroids or hospital admissions in the past year should be treated as patients with uncontrolled asthma, even if the level of control seems good in between the exacerbations (**Evidence D**).

### Treatment steps for asthma control (Steps 2 to 5)

The following are recommended for each step:

### Treatment at Step 2:

- The preferred recommendation for step 2 level is daily ICS at a low dose (< 500 μg of beclomethasone equivalent/day [Table 3] (**Evidence A**).[59–123,124]
- Alternative treatments include LTRA (montelukast) (**Evidence A**).[125]

### Treatment at Step 3:

- Adding a LABA to a low-medium dose ICS, such as Fluticasone/Salmeterol (Seretide) or Budesonide/Formoterol (Symbicort) for patients whose asthma is not controlled on a low dose ICS alone improves asthma control (**Evidence A**).[126,127,128] The standard strategy is to use a maintenance dose of the combination drugs twice daily and use the rapid onset B2-agonist as a reliever treatment (**Evidence A**).[129]
- If a combination inhaler containing Formoterol/Budesonide is selected, patient may be advised to use it for both rescue and maintenance i.e., S.M.A.R.T ^®^ approach (**Evidence A**).[130] At this step of care, maintenance dose of Budesonide/Formoterol single inhaler (1-2 puff 160/4.5 BID) is selected plus extra puffs from the same inhaler up to a total of 12 puffs per day. Those patients who require such high dose should seek medical advice to step up therapy that may include use of short course of oral prednisone.[131]
- Alternative but in general less effective strategies include: the continuation of ICS as a monotherapy by increasing the dose to the medium to high dose range (**Evidence A**),[131,132] the addition of LTRA to a low-medium dose ICS (**Evidence A**),[133,134] especially in patients with concomitant rhinitis.[135] The addition of sustained release theophylline to a low-medium dose ICS is a third alternative choice (**Evidence B**).[136]
- GOAL study has shown that an escalating dose of combination of Fluticasone/ Salmeterol (Seretide) achieves well controlled asthma in 85% of patients and totally controlled asthma in 30% (**Evidence B**).[137]
- Consultation with a specialist is recommended for patients whenever there is a difficulty in achieving control (**Evidence D**).

### Treatment at Step 4:

- Consultation with a specialist is recommended for patients who require this step of therapy (**Evidence D**).
- At this level of care, maximizing treatment is recommended by combining high-dose ICS with LABA (**Evidence B**).[104,132,133,138]
- Adding LTRA or theophylline to high-dose ICS and LABA should be considered (**Evidence D**).
- Omalizumab may be considered at this step for patients who have allergic asthma (as determined by skin test or RAST study) and still uncontrolled (**Evidence A**). Special knowledge about the drug and its side effects should be available before administering Omalizumab by any physician.

### Treatment at Step 5:

- Consultation with an asthma specialist is recommended to be mandatory for patients who require this step of therapy (**Evidence D**).
- In patients who continue to be symptomatic despite step 4 level of care, omalizumab to be considered at this step for patients who have allergic asthma and persistent symptoms despite the maximum therapy mentioned above (**Evidence A**).[104,132,133,139]
- If the patient does not have allergic asthma or omalizumab is not available or not adequately controlling the disease, the alternative approach is to use the lowest possible dose of long-term oral corticosteroids (**Evidence D**).
- For patients who require long-term systemic corticosteroids, the following should be considered:
  - Use the lowest possible dose to maintain control.
  - Closely monitor for the development of corticosteroid-related side effects.
  - When controlled asthma is achieved, continue attempts to reduce the dose of systemic corticosteroids. Maintaining high-dose of ICS therapy may help to reduce the dose of systemic steroid.
  - Adjustment of steroid dose at the time of stress (for example, infection, exacerbation, surgery, etc.) is essential.
  - Strongly consider concurrent treatments with calcium supplements, vitamin D, and bone-sparing medications (for example, bisphosphonates) in patients who have risk factors for osteoporosis or low bone mineral density (**Evidence C**).[93]

### Special consideration for asthma treatment in children younger than five years

To date, there is no clinically validated objective asthma control measure for a child younger than five years [Table 8]. Level of control is based mainly on symptoms recognized by the child's caregiver and the need for rescue treatment. In this age group, the following are recommended:

**Table 8:** Levels of asthma control for children 5 years and younger

Component of control	Classification of asthma control		
	
	Controlled	Partially controlled	Uncontrolled
Symptoms	≤2 days/week	>2 days/week	Throughout the day
Nighttime awakenings	Once a month	>once a month	>once a week
Effect on daily activities	None	Some limitations	Extremely limited
Rapid-onset B2-agonist for symptoms relief	≤2 days/week	>2 days/week	Several time/day

Adopted from GINA report on asthma in children 5 years and younger

- The most effective bronchodilator available is SABA that can be delivered as needed by MDI and spacer (**Evidence A**).[95,140,141,142]
- If control is not achieved and controller treatment commenced, the lowest dose of ICS delivered by MDI and a spacer is recommended (**Evidence A**).[143] LTRA is considered as an alternative therapy especially when there is concomitant rhino-sinusitis.[144,145]
- If asthma control is not achieved on low dose ICS, doubling the initial dose is recommended (**Evidence C**).[146]
- If further control is needed, ICS dose can be increased to the maximum, and/or adding an LTRA or theophylline (**Evidence D**).
- Low dose of oral corticosteroids for a few weeks to achieve control should be limited to severe uncontrolled cases to avoid the side effects from corticosteroids (**Evidence D**).
- For children with seasonal symptoms, daily controller therapy may be discontinued after the season with the advice for a follow-up visit within 3-6 weeks (**Evidence D**).
- Frequent episodes induced by severe viral infection may justify a diagnostic trial of regular controller therapy to confirm whether the symptoms are due to asthma (**Evidence D**).

### Immunotherapy

Allergen immunotherapy (AIT) is the gradual immunization process in which increasing doses of standardized allergen responsible for causing allergic symptoms are repeatedly administered to patients with IgE- mediated allergic diseases either subcutaneously or sublingually.[147] This will induce increased tolerance to the allergen that may provide long-term relief of symptoms during subsequent exposure to the same allergen. AIT is more effective in seasonal asthma than in perennial asthma, particularly when used against a single allergen. AIT may be considered if strict environmental avoidance and comprehensive pharmacologic intervention by an asthma specialist have failed to control asthma (**Evidence B**).[148] Their side effects include systemic allergic reactions, occasional anaphylaxis and, even, rare fatalities. Therefore, any anticipated benefits must be weighed against the risk of adverse effects, cost, and inconvenience of the prolonged course of therapy.

## Special Situations

### Asthma and pregnancy

Asthma is present in up to 8% of pregnant women. The course of asthma during pregnancy is unpredictable; however, one third of pregnant asthmatics will have worsening of their asthma control.[149,150] Maintaining adequate control of asthma during pregnancy is essential for the health and well-being of both the mother and her baby. Identifying and avoiding triggering factors should be the first step of therapy for asthma during pregnancy. Treatment should take the same stepwise approach as in the nonpregnant patient. Salbutamol is the preferred SABA because it has an excellent safety profile. ICS are the preferred treatment for long-term control (**Evidence B**).[151] Use of ICS, theophylline, antihistamines, B2-agonists, and LTRA is generally safe and has not been shown to increase the risk of foetal abnormalities.[152] Prolonged use of systemic steroids may be associated with pregnancy related complications. Acute exacerbations of asthma during pregnancy should be treated on the same outlines as in nonpregnant patients.[153]

### Cough-variant asthma

Patients with cough-variant asthma have chronic cough as their main, if not the only symptom.[154] It is particularly common in children, and is often more problematic at night. Other diagnoses to be considered are drug-induced cough caused by angiotensin-converting-enzyme inhibitors, GERD, postnasal drip and chronic sinusitis. Treatment should follow the same stepwise approach for the long-term management of asthma.[155]

### Exercise-induced asthma

Asthma-like symptoms can sometimes be triggered only by physical activities. Normally, bronchodilatation occurs during exercise and lasts for a few minutes afterward. In patients with exercise-induced asthma, the initial bronchodilatation is followed by bronchoconstriction that generally peaks within 10 to 15 minutes after completing the exercise and resolves within 60 minutes. EIB can be prevented by use of rapid acting B2-agonist few minutes before exercise (**Evidence A**).[156] Warm-up period before exercise may reduce EIA. If this approach does not control the symptoms patients should have maintenance therapy with ICS increased or introduced (**Evidence A**). Regular use of LTRA may help in this condition especially in children (**Evidence B**).[157,158]

### Aspirin-induced asthma

About 10-20% of adults with asthma suffer from exacerbations in response to aspirin or NSAIDs, which is more common in severe asthma. The majority of patients experience first symptoms during the third to fourth decade. Once aspirin or NSAID hypersensitivity develops, it is present for life. Characteristically, within minutes to one or two hours following ingestion of aspirin, an acute, severe attack develops, and is usually accompanied by rhinorrhea, nasal obstruction, conjunctival irritation, and scarlet flush of the head and neck.[159] A typical history of reaction is considered adequate for diagnosis of aspirin induced asthma. Patients known to have aspirin induced asthma should avoid all aspirin-containing products and NSAIDs. Where an NSAID is strongly indicated, alternative analgesics such as paracetamol should be considered. Prophylactic low-dose aspirin should also be avoided; however, patients for whom aspirin is considered essential, they should be referred to an allergy specialist for aspirin desensitization. Aspirin and NSAID can be used in asthmatic patients who do not have aspirin induced asthma.[160]

### Gastro-esophageal reflux disease triggered asthma

Gastro-esophageal reflux disease is more prevalent in patients with asthma compared to the general population. The mechanisms of GERD triggered asthma includes vagal mediated reflex and reflux secondary to micro-aspiration of gastric contents into the upper airways.[161] All patients with asthma should be questioned about symptoms of GERD. If symptoms presents, a trial of GERD therapy, consists of a high-dose proton pump inhibitor with motility agent for 6-12 weeks and lifestyle modifications, may be considered (**Evidence B**).[162] Asymptomatic patients with uncontrolled asthma may not benefit from GERD therapy (**Evidence B**).[163]

## Management of Acute Asthma

**Figure 5:**
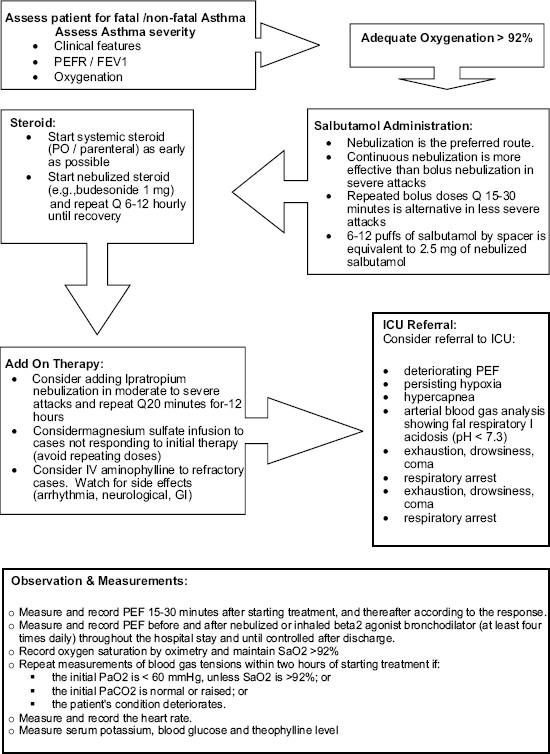
Algorithm of acute asthma management

Most patients who presented with an acute asthma exacerbation have chronic uncontrolled asthma.[164] Many of the deaths have been reported in patients who have received inadequate treatment or poor education. The following should be carefully checked: previous history of near fatal asthma, patient of three or more medications, heavy use of SABA, repeated visits to emergency department, and brittle asthma. Upon presentation, a patient should be carefully assessed to determine the severity of the acute attack and the type of required treatment [Table 9].[165,166] PEF and pulse oximetry measurements are complementary to history taking and physical examination [Figure 5].

**Table 9:** Levels of severity of acute asthma exacerbations

Near fatal asthma	Raised PaCO_2_ and/or requiring mechanical ventilation
Life threatening asthma	Any one of the following in a patient with severe asthma:
	PEF <33% best or predicted
	Bradycardia
	SpO_2_ <92% (PaO_2_ <60 mmHg) on high flow FIO_2_
	Cyanosis
	Dysrhythmia
	Hypotension
	Normal or high PaCO_2_
	Exhaustion
	Confusion
	Silent chest
	Coma
	Weak respiratory effort
Acute severe asthma	Any one of:
	PEF 33–50% best or predicted
	Respiratory rate ≥25/min
	Heart rate ≥ 110/min
	Inability to complete sentences in one breath
Moderate asthma exacerbation	Increasing symptoms
	PEF 50–75% best or predicted
	No features of acute severe asthma
Brittle asthma	Type 1: Wide PEF variability (>40% diurnal variation for >50% of the time over a period >3–6 months) despite intense therapy
	Type 2: Sudden severe attacks on a background of apparently well controlled asthma

*Treatment of acute asthma in adults and children older than 5 years Oxygen:* High concentration of inspired oxygen should be used to correct hypoxemia. However, pulse oximetry should be used to tailor oxygen therapy. Failure to achieve oxygen saturations more than 92% is a good predictor of the need for hospitalization (**Evidence C**).[167] Normal or high PaCO_2_ is an indication of a severe attack, and the need for specialist consultation.[168]

*Bronchodilators:* In acute asthma, inhaled salbutamol is the preferred choice (**Evidence A**). Repeated doses should be given at 15-30 minute intervals. Alternatively, continuous nebulization (Salbutamol at 5-10 mg/hour) may be used for one hour if there is an inadequate response to initial treatment. In patients who are able to use the inhaler devices, 6-12 puffs of MDI with a spacer are equivalent to 2.5 mg of Salbutamol by nebulizer.[168] In moderate to severe acute asthma, there is some evidence to show that combining ipratropium bromide with Salbutamol has some additional bronchodilation effects (**Evidence B**), in reducing hospitalizations (**Evidence A**) and greater improvement in PEF or FEV_1_ (**Evidence A**).[169,170,171]

*Steroid therapy:* Except for mildest asthma execrations, systemic steroids have been proven to reduce relapses, subsequent hospital admission and requirement for B_2_ -agonist therapy (**Evidence A**).[172] Oral steroid are as effective as injected steroids, provided tablets can be swallowed and retained and a patient does not become drowsy or vomiting. For adults patients, doses of oral prednisolone of 40-60 mg daily or parenteral steroids (intravenous hydrocortisone 300-400 mg in divided doses or total of 60-80 mg of intravenous methylprednisolone are adequate for hospitalized patients (**Evidence B**).[173] Systemic steroids should be given for seven days for adults and three to five days for children (**Evidence B**).[173] However, recent data showed that early addition of ICS can benefit patients in acute asthma and can reduce the amount of oral steroid after hospital discharge and lower rate of relapse (**Evidence B**).[174]

*Intravenous magnesium sulphate:* A single dose of IV magnesium sulphate (1.2-2 gm IV infusion over 20 mins) has been shown to be safe and effective.[175] However, routine use of IV magnesium sulphate in patients with acute asthma presenting to emergency department is not recommended.[176] Its use should be limited to those with sever exacerbation who fail to respond to treatment after an hour.

*Intravenous aminophylline:* In acute asthma, the use of intravenous aminophylline did not result in any additional bronchodilation compared to standard care with B2-agonists (**Evidence A**).[177]

*Antibiotics:* Viral infection is the usual cause of asthma exacerbation. The role of bacterial infection has been probably overestimated, and routine use of antibiotics is strongly discouraged. They should be used when there is associated pneumonia or bacterial bronchitis.

*Referral to a specialist center:* Indications for referral to a specialist center or admission to the intensive care area include patients with the following: status asthmatics, deteriorating PEF, persisting or worsening hypoxia, increasing hypercapnea, respiratory acidosis (pH < 7.3), severe exhaustion and increase work of breathing, drowsiness and confusion, coma or respiratory arrest (**Evidence D**).[178]

*Criteria for admission:* The patient has to be admitted when he shows any feature of a life threatening, near fatal attack, or any feature of a severe attack that persists after initial treatment. Patients whose peak flow is ≥60% best or predicted one hour after initial treatment can be discharged from the emergency department unless any of the following is present: still suffering from significant symptoms, previous history of near fatal or brittle asthma, concerns about compliance (living alone, social, education or psychological problems, etc.), or pregnancy.

### Special consideration for acute asthma treatment in children younger than five years

An acute exacerbation of asthma in children of five years and below is defined as an acute or sub-acute deterioration in symptom control that is sufficient to cause distress or risk to health necessitating a visit to a health care provider or requiring treatment with systemic corticosteroids.[6]

Early symptoms of an acute exacerbation would usually follow an upper respiratory infection. The symptoms include: shortness of breath, wheeze, nocturnal cough, exercise indolence. Initiation of treatment at home with two puffs (200 μg) of salbutamol via spacer is recommended (**Evidence D**). Immediate medical attention should be taken in case of children less than two year who had a history of poor response to three doses of SABA within 1-2 hours, saturation less than 92%, or the child is acutely distressed. In this age group, the risk of fatigue, respiratory compromise and dehydration is considerable [Table 10].

**Table 10:** Initial management of acute severe asthma in children of five years and below

Therapy	Dose and Administration
• Supplemental oxygen to maintain O_2_ Saturation >94%	Deliver 24% by face mask
• Short-acting B2-agonist	2–4 puffs by spacer and mask or 2.5 mg salbutamol by nebulizer
	Repeat every 20 minutes for first hour
• Ipratropium bromide	2 puffs or 125 micrograms by nebulizer every 20 minutes for the first hour
• Systemic corticosteroids	Oral prednisolone (1–2 mg/kg for 1–5 days)
	Intravenous methylprednisolone 1 mg/kg 12 every 6 hours on day 1; every 12 hours on day 2, than once
